# 766 Evaluating the Ethics of Comfort Care in Self-Immolation Burns

**DOI:** 10.1093/jbcr/irad045.241

**Published:** 2023-05-15

**Authors:** Theophilus Pham, Sharmila Dissanaike, Kripa Shrestha, Chathurika Dhanasekara

**Affiliations:** TTUHSC, Lubbock, Texas; TTUHSC, Lubbock, Texas; TTUHSC, Lubbock, Texas; TTUHSC, Lubbock, Texas; TTUHSC, Lubbock, Texas; TTUHSC, Lubbock, Texas; TTUHSC, Lubbock, Texas; TTUHSC, Lubbock, Texas; TTUHSC, Lubbock, Texas; TTUHSC, Lubbock, Texas; TTUHSC, Lubbock, Texas; TTUHSC, Lubbock, Texas; TTUHSC, Lubbock, Texas; TTUHSC, Lubbock, Texas; TTUHSC, Lubbock, Texas; TTUHSC, Lubbock, Texas

## Abstract

**Introduction:**

Self-Immolation injuries, although rare, incur a high mortality rate due to the high injury burden. These patients often require a long hospital stay including extended periods of time in the ICU due to the systemic burden of their burns. They often present with active suicidal ideation leaving medical decisions to be made by their next of kin who want to see everything done to preserve the life of their loved ones. Over the past several years our burn center has taken a more holistic approach to the care of these patients involving both the psychiatry team as well as the palliative care team to discuss concurrent treatment and prognosis with both patients and their families. Previous ICU dogma often discourage earlier palliative care consultation as many providers and families see such actions as the care team no longer wishing to provide care for patients

**Methods:**

Single patient case study and review

**Results:**

The early consultation of a supportive care team allowed the patient's family more access to resources to better make a better informed decision that was in keeping with the patient's wishes

**Conclusions:**

Patients who commit self immolation often present with active suicidal ideations and are deemed unfit to make their own medical decisions. It is of utmost importance to not only treat their burns but also for early psychiatric treatment of their underlying condition when possible. These patients often are not coherent or are determined to not have the autonomy to make their own medical decision. Until the patient is deemed capable of making their own medical decision, it is up to the providers to determine a surrogate decision maker. The role of the surrogate decision maker is difficult as they must use substitute judgment in order to make decisions based on the subjective wishes of the patient. The difficulty in this situation arises from the fat that the decisions although made based upon the patient’s wishes can be at conflict with the patient’s best interest. When these conflicts arise it is up to medical providers to have an open dialogue and convey all revelevent information related to prognosis and outcome with the decision maker. There have been no studies that look at the ethics of palliative care among these patients and how to best honor their wishes.

**Applicability of Research to Practice:**

This study is to help foster a discussion around the ethics of early discussion of palliative measures in regards to the treatment of self-immolation burns

